# Platelets in Hepatocellular Carcinoma—From Pathogenesis to Targeted Therapy

**DOI:** 10.3390/cancers17142391

**Published:** 2025-07-18

**Authors:** Natalia Kluz, Hanna Grabowska, Paulina Chmiel, Kornelia Rynkiewicz, Alicja Skrobucha, Ewa Wysokińska, Łukasz Szymański, Piotr Tomasz Wysocki, Aleksandra Semeniuk-Wojtaś, Leszek Kraj

**Affiliations:** 1 Medical Faculty, Medical University of Warsaw, 02-091 Warsaw, Poland; natalia.kluz@wum.edu.pl (N.K.); rynkiewiczkornelia@gmail.com (K.R.); 2 Faculty of Medicine, Cardinal Stefan Wyszynski University, 5, 01-815 Warsaw, Poland; grabowska.hanna@icloud.com; 3 Department of Soft Tissue/Bone Sarcoma and Melanoma, Maria Sklodowska-Curie National Research Institute of Oncology, 00-001 Warsaw, Poland; 4 Department of Cardiology, University Clinical Centre, Medical University of Warsaw, 02-091 Warsaw, Poland; alicja.skrobucha@wum.edu.pl; 5 Division of Hematology and Medical Oncology, Mayo Clinic, Jacksonville, FL 32224, USA; wysokinska.ewa@mayo.edu; 6 Department of Molecular Biology, Institute of Genetics and Animal Biotechnology, Polish Academy of Sciences, 01-447 Jastrzębiec, Poland; l.szymanski@igbzpan.pl (Ł.S.); leszek.kraj@wum.edu.pl (L.K.); 7 Department of Oncology and Radiotherapy, Medical University of Gdańsk, 80-214 Gdańsk, Poland; ptwysocki@gumed.edu.pl; 8 Department of Oncology, University Clinical Centre, Medical University of Warsaw, 02-091 Warsaw, Poland; aleksandra.semeniuk-wojtas@wum.edu.pl

**Keywords:** hepatocellular carcinoma, HCC, platelets, PLT, liver cancer, biomarkers

## Abstract

Hepatocellular carcinoma (HCC) is a malignancy with a complex pathogenesis, course, and prognosis with increasing incidence. The most significant contributing factor to the development of HCC is the chronic process of inflammation and remodeling of the cirrhotic liver, in which the interaction between the tumor microenvironment (TME) and cancer cells plays a pivotal role. In recent years, increasing focus has been directed toward the role of platelets (PLTs) in mediating interactions between tumor cells and the TME and in the progression and spread of HCC, as well as other cancers. Due to their abundance in the bloodstream and intracellular granules rich in mediators facilitating their ability to modulate the immune system, PLTs play a significant role in carcinogenesis. In the context of HCC, the role of PLTs in the healing and regeneration processes of the liver has been recognized for some time. In recent years, there has been an increasing utilization of PLTs in prognostic models for patients with HCC. Given their role and the availability of clinical options that block PLTs’ action, clinical trials of platelet blockers in the adjunctive treatment of HCC are becoming increasingly common. However, further research, both preclinical and clinical, is necessary to fully elucidate the role of PLTs in HCC and their potential use as a therapeutic target. In this literature review, we summarize the current knowledge on PLTs in HCC and focus on their potential use in everyday clinical practice.

## Simple Summary

Platelets are increasingly recognized as key contributors to the development and progression of hepatocellular carcinoma (HCC), the most common type of liver cancer. Traditionally known for their role in blood clotting, platelets are now recognized as active players in tumor development, progression, and metastasis. They release factors that promote inflammation, tumor growth, angiogenesis, and immune evasion. Platelet counts are increasingly used in prognostic models for HCC, with both high and low levels linked to poor outcomes. Recent studies suggest that antiplatelet therapies like aspirin may help prevent HCC or improve treatment outcomes. However, bleeding risks in cirrhotic patients remain a concern. This review highlights the need for further research, especially clinical trials, to assess the therapeutic potential of targeting platelets in HCC treatment.

## 1. Introduction

Approximately 865,000 new cases and 664,000 deaths worldwide were reported due to hepatocellular carcinoma (HCC) in 2022 [1]. HCC comprises 75%–85% of primary liver cancer cases and is the sixth-most common cancer type and the third-leading cause of cancer-related mortality globally [2]. It is generally assumed that the highest number of cases of this cancer occurs annually in Africa and Asia, which correlates with the geographical distribution of viral hepatitis B (HBV) and viral hepatitis C (HCV), the two main causes of progressive chronic liver disease and HCC [3]. In highly developed countries, nonalcoholic fatty liver disease (NAFLD) plays a major role in the development of HCC, with the significant increase in the prevalence of obesity and type 2 diabetes in recent years contributing to this trend [4,5]. HCC typically develops as a result of chronic liver damage and persistent inflammation [6], with cirrhosis being a major risk factor, regardless of its etiology [7,8,9]. Up to 90% of HCC cases are characterized by chronic inflammation, fibrosis, and/or liver cirrhosis [10]. Consequently, the prognosis of patients with HCC is closely dependent on the functional status and health of the liver. HCC often presents with non-specific symptoms in the early stages, which hinders the ability to make a timely diagnosis when liver function is preserved, complicating therapeutic options [11].

The pathogenesis and progression of HCC is a complex process mediated partly by two major cell types, stellate cells and Kupffer cells. Activation of Kupffer cells results in the secretion of various mediators, including cytokines and chemokines, which in turn determine immune responses, inflammation, and the recruitment of other liver cells [12]. Stellate cells become activated upon injury and transform into a phenotype similar to activated myofibroblasts [13]. These processes lead to the accumulation of extracellular matrix (ECM) proteins, the release of cytokines and patterning factors, and the generation of reactive oxygen species (ROS). Consequently, those cells generate a proinflammatory tumor microenvironment (TME), which in turn contributes to the further excessive stiffening of the cirrhotic liver [14]. Furthermore, in HCC, tumor and TME cells interact with components of the hemostatic system via impaired blood vessels, activating the coagulation cascade and fibrinolytic processes and altering liver hemodynamics. These changes promote cellular proliferation and tumor growth [15].

Platelets (PLTs) are morphologically distinct cells produced in the bone marrow with a primary hemostatic role [16] of initiating clot formation in response to vascular injury [17]. Upon adhesion to the ECM via von Willebrand factor, they become activated via collagen receptor GPVI, leading to aggregation and ultimately thrombus formation [18]. During the activation phase, platelets release α-granules and dense granules, which contain proinflammatory cytokines, chemokines, and growth factors, including platelet-derived growth factor (PDGF), serotonin, endothelial growth factor (EGF), insulin-like growth factor (IGF-1), transforming growth factor beta (TGFβ), tumor necrosis factor alpha (TNF-α), interleukin 6 (IL-6), chemokine (C-X-C motif) ligand 4 (CXCL4), vascular endothelial growth factor A (VEGF-A), hepatocyte growth factor (HGF), and fibroblast growth factor (FGF) [19]. These mediators are involved in the wound healing process under normal physiological conditions; however, it has also been found that these factors can negatively affect the TME [20].

Platelets play an important role in tissue regeneration and are actively recruited in liver injury, where they stimulate hepatocyte proliferation by releasing high concentrations of serotonin [21]. In the context of HCC and its complex TME, PLTs appear to fulfill a multitude of functions beyond their direct impact on hepatocyte proliferation. They engage in numerous interactions with TME cells, contributing to a complex and dynamic interplay. The available evidence suggests a direct influence of platelet-derived factors on tumor cell proliferation, as well as pro-fibrinogenic signaling and immune cell recruitment, and a mediating effect between these different processes within the TME [20,21].

Finding novel therapeutic strategies for HCC remains a complex challenge due to the tumor’s high heterogeneity, immunosuppressive microenvironment, and frequent resistance to standard therapies. Current treatments, such as tyrosine kinase inhibitors and immune checkpoint inhibitors, often yield limited and temporary responses, especially in advanced stages [22,23]. A major limitation lies in the TME, which actively supports tumor survival and impairs immune-mediated clearance [24]. In this context, targeting PLTs has emerged as a promising strategy. PLTs contribute to tumor progression by modulating immune responses, promoting angiogenesis, and protecting circulating tumor cells. Therapeutic interventions aimed at inhibiting platelet activation or blocking platelet-derived signaling pathways hold potential to disrupt these tumor-promoting mechanisms and enhance the efficacy of existing therapies in HCC. The objective of this review is to provide a comprehensive definition of the function of PLTs in the pathogenesis and progression of HCC and to discuss their role in the context of interaction with the TME. Particular focus has been dedicated to translating the pathophysiology into the potential utilization of PLTs in HCC clinical practice, both as biomarkers and as targets for modern therapies in clinical trials.

## 2. Platelets: A Brief Overview

**Figure 1:**
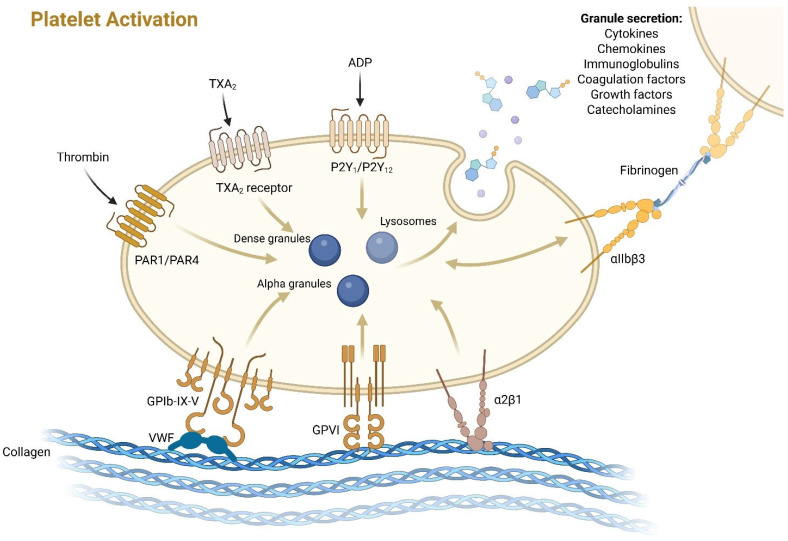
Schematic representation of platelet activation (PLT). The GPIb-IX-V complex, GPII-IIIa, and α2β1 receptors respond to collagen and vWF exposed on damaged endothelium. Activation of PLTs receptors can also occur via ADP binding to the P2Y1 receptor or TXA2 to its receptor. Another potent activating signal is the binding of thrombin to the PAR surface receptors. Following a complex signaling cascade from α-granules, dense granules, and lysosomes, signaling molecules (mediators) are released into the environment. These molecules induce both vascular repair and inflammatory processes in the area of damage. Integrin αIIbβ3 is a critical mediator of platelet aggregation. ADP—adenosine diphosphate; GPIb-IX-V—glycoprotein Ib-IX-V; GPII-IIIa—glycoprotein PII-IIIa; PAR1/4—protease-activated receptor 1/4; P2Y1/12—purinergic receptor 1/12; TXA2—thromboxane A2; vWF—von Willebrand factor.

Platelets are anucleate blood cells (2–4 μm in diameter) that arise from megakaryocytes primarily in the bone marrow and circulate in blood for 7–10 days, after which they are eliminated in the spleen and liver [16]. They are characterized by the presence of open canalicular systems and an intricate array of membranes that facilitate communication between its α-granules, dense granules, lysosomes, and mitochondria with the extracellular space [25]. This intricate network of membrane-to-membrane communication is crucial for the fulfillment of basic physiological functions by platelets, thereby contributing to the maintenance of homeostasis within the body. In the absence of activation stimuli, platelets persist in the bloodstream as disks bearing numerous surface receptors [26,27]. These receptors are responsible for platelet transformation into a more spherical, active form [28]. This active form releases mediators that facilitate coagulation, intercellular communication, and inflammatory processes (Figure 1). The most significant of these receptors are the glycoproteins (GPs), which include the GPIb-IX-V complex responsible for platelet adhesion and the GPII-IIIa receptor, which facilitates platelet aggregation [29]. The process of activation and degranulation is multi-stage and complicated, mediated via numerous receptors, including purinergic receptor (P2Y 1), adenosine diphosphate (ADP), and thromboxane A2 (TXA2) receptors, which results in increased intracellular calcium ion (Ca^2+^) level and degranulation. Thrombin is the strongest agonist capable of activating platelets through interacting with protease-activated receptors (PARs) on their surface [30]. Coagulation factors, adhesion proteins (e.g., fibrinogen, fibronectin, and von Willebrand factor), chemokines (whose functions will be discussed in the following sections), immunoglobulins, protease inhibitors (e.g., C1 inhibitor, plasminogen activator inhibitor 1, and platelet factor XIα inhibitor), and amines (e.g., adrenaline, noradrenaline, and serotonin) are released from intracellular reservoirs [31]. These processes ultimately result in the formation of a thrombus, which then causes the vessel damage.

Apart from secreting substances necessary for maintaining homeostasis, activated PLTs also interact with neutrophils, monocytes, and lymphocytes [32,33,34]. It is imperative to note that P-selectin, via its ligand, P-selectin glycoprotein ligand-1 (PSGL-1), plays a pivotal role in these interactions, particularly within the context of inflammatory processes [35]. This interaction contributes to the establishment of a proinflammatory environment by further stimulating the release of proinflammatory cytokines. Furthermore, activated platelets express functional CD40 ligand (CD40L), also known as CD154. CD40L is a transmembrane molecule that plays a crucial role in cell signaling in both innate and adaptive immunity [36].

Key mediators, vital for both physiological processes and systemic diseases, released by platelets are summarized in Table 1.

## 3. Platelets in HCC Pathogenesis and Metastases

Studies on the role of PLTs in the progression of HCC emphasize their function as active mediators in the TME. Key roles include their profibrogenic signaling, mediation of proinflammatory responses, and promotion of cell proliferation, all of which may contribute to a more aggressive course of HCC [37]. Interactions between hepatocytes and platelets via IGF-1-mediated pathways provided a strong proliferative signal [38]. Recent studies have centered on elucidating this phenomenon, which is predominantly attributed to the release of substances by platelets and their surface receptors. He et al. demonstrated that *Krüppel-like factor 6* (*KLF6*), considered one of the suppressor genes in HCC, is suppressed by substances released from PLTs. Furthermore, a reduced percentage of apoptosis in HCC cells and an increased population of HCC cells in the S and G2/M phases of the cell cycle, as well as a reduced cell population in the G0/G1 phase, were observed [39]. Moreover, a subsequent study demonstrated that platelets can exert a proliferative effect on liver cells through interaction with Kupffer cells, thereby facilitating the most potent stimulation of regeneration [40,41]. Serotonin has been identified as a significant factor in the proliferation of HCC [42]. The primary storage location of serotonin in the bloodstream is the dense granules of platelets. It has been demonstrated that serotonin exerts a proliferative effect on HCC cells via the transcription factor FOXO3a, among others [43]. Furthermore, HCC cells exhibit increased expression of serotonin receptors. Consequently, the signal transmitted by serotonin enables these cells to evade proapoptotic signals [42].

Platelets strongly influence tumor cell migration by adhering to cancer cells via receptors such as GPIIb/IIIa, GPIb-IX-V, and P-selectin [44]. Although the role of PLTs in metastatic spread has been studied [45], limited information is available regarding their significance in HCC. It has been shown that in patients with HCC, the platelet count is higher in those with extrahepatic metastases compared to patients without metastases, suggesting a role for platelets in metastasis formation [46]. Furthermore, PLTs aggregate with circulating tumor cells (CTCs) in the vasculature, protecting them from shear stress and immune surveillance [47].

When considering the mechanisms of cancer dissemination, epithelial-to-mesenchymal transition (EMT) is a fundamental process by which tumor cells gain invasiveness and enter the bloodstream, ultimately leading to extravasation and metastasis [48]. It is important to note that the primary triggering factors are the tumor cells that invade the bloodstream and migrate. Tumor cells can release mediators, leading to platelet activation, which in turn can promote cancer progression and metastasis in addition to further enhancing platelet activation [49].

Transforming growth factor beta 1 (TGFβ1) stored in platelet α-granules is an important initiator of tumor cell proliferation. It can cross blood vessel walls and diffuse into the tissue microenvironment, thus creating the TME and promoting tumor cell invasion [50]. It has been shown that blocking TGFβ signaling reduces the suppression of KLF6 expression by PLTs [51]. This effect was absent when KLF6 was silenced in HCC cell lines or when HCC cells were incubated with platelets devoid of secreted substances under physiological conditions [39]. This confirms that platelet-derived TGFβ initiates a TGFβ/Smad and NF-κB signaling cascade in tumor cells, enabling EMT and promoting metastasis formation [52].

**Figure 2:**
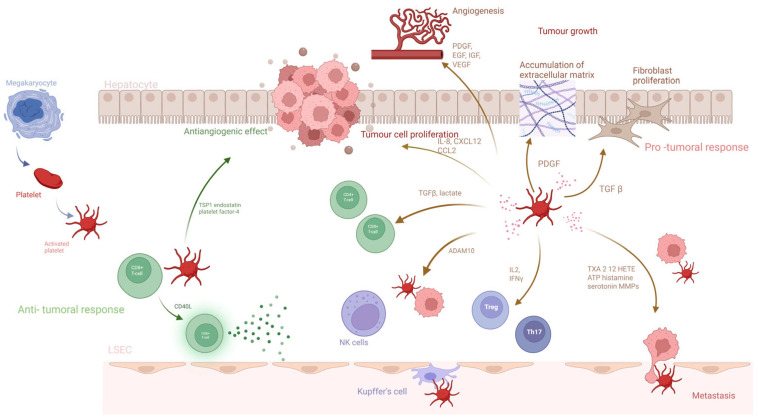
Schematic diagram illustrating the multifaceted role of platelets in hepatocellular carcinoma (HCC) progression and metastasis. Platelets take part in both antitumoral and pro-tumoral responses. Platelets, through CD40L, activate CD8+, and through the activity of TSP1, endostatin, and platelet factor 4 contribute to the antitumoral response. However, the pro-tumoral response is much more evident. Platelets contribute to tumor growth by influencing tumor cell proliferation (IL-8, CXCL12, and CCL2), accumulation of extracellular matrix (PDGF), and fibroblast proliferation (TGFβ). Moreover, platelets inhibit the immune response. TGFβ and lactate inhibit CD4+ and CD8+ T-cell activity, and IL-2 and IFNγ promote T-reg and Th17 lymphocytes, thus additionally weakening the immune response. Tumor cells, through interacting with platelets using ADAM10, induce shedding of NKG2P ligands, such as MICA/B and ULBP2, on their cells, which weakens immune recognition and the NK cells. Platelets activated by tumor cells, through TXA2, 12HETE, ATP, histamine, and serotonin, induce endothelial cell retraction. Moreover, MMPs and platelet hyaluronidase 2 heparin lead to the degradation of extracellular matrix components and basement membrane degradation. Both endothelial cell retraction and basement membrane degradation lead to HCC metastasis. Important to note is the presence of Kupffer cells, which, through interacting with platelets, induce inflammatory processes leading to cirrhosis and the secondary development of HCC.

Platelets can promote EMT in both primary tumors and metastases of HCC through autophagy induced in HCC cells by TGFβ1 via the AMPK/mTOR pathway [53]. Via RNA sequencing, it was found that platelets release TGFβ1 and alter the signaling pathway in tumor cells. Inhibition of TGFBR or removal of platelet-derived TGFβ1 led to suppression of AMPK/mTOR pathway activation and autophagy in response to platelet release. As a result, there was a reduction in EMT, decreased autophagy, and lower tumor cell migration and metastasis formation. Additionally, inhibiting autophagy by reducing Atg5 in tumor cells prevented EMT and metastasis formation in the context of platelet-derived TGFβ1 [53]. Moreover, platelet count directly correlated with increased expression of TGFβ1, LC3, and N-cadherin in primary tumors of HCC patients [53]. This suggests a clear association between platelet-dependent progression in HCC (Figure 2).

## 4. Role of Platelet-Derived Mediators in HCC

In addition to their well-established physiological roles in hemostasis and maintaining vascular endothelial integrity, platelets are multifunctional cells that are also actively involved in tumor growth, angiogenesis, and distant metastasis [54,55,56,57]. A large number of platelets are present in the TME, where they contribute to tumor progression by releasing platelet-derived microparticles (PMPs) that support tumor overgrowth [58,59,60].

Platelets possess a plethora of growth factors, including TGFβ and basic fibroblast growth factor (bFGF) [61]. Their α-granules and dense granules store a variety of proteins, including cytokines such as interleukin 1α (IL-1α), IL-1β, and TGFβ1, as well as chemokines like chemokine (C-X-C motif) ligand (CXCL) 1/growth-regulated oncogene α (GRO-α), CXCL4, CXCL5 (epithelial cell-derived neutrophil-activating peptide (ENA-78)), CXCL7, CXCL8 (IL-8), CXCL12 (stromal cell-derived factor 1 (SDF-1)α, CCL2 (monocyte chemoattractant protein 1 (MCP-1), CCL3 (macrophage inflammatory protein 1 (MIP-1α)), and CCL5 (regulated on activation, normal T-cell expressed and secreted (RANTES)), as well as immunomodulatory neurotransmitters, including serotonin (5-hydroxytryptamine (5-HT), dopamine, epinephrine, histamine, and gamma-aminobutyric acid (GABA), and other low-molecular-weight mediators [62,63]. Upon activation, platelets release various factors, such as PDGF, EGF, IGF, and VEGF, which collectively promote tumor growth and stimulate angiogenesis [64]. In vitro studies have demonstrated that these growth factors enhance cancer cell proliferation in a dose-dependent manner, whereas in vivo studies indicate that circulating platelets release these factors into the bloodstream upon activation, thereby enhancing the intravascular proliferation of cancer cells [65]. Cancer-associated fibroblasts (CAFs), a major component of the TME, play essential roles in tumor development. Platelet-derived PDGF signaling can activate CAFs, which in turn secrete factors that influence platelet function. PDGF B released from platelets enhances CAF accumulation and extracellular matrix deposition [66]. Additionally, TGFβ secreted by platelets enhances fibroblast proliferation, further contributing to tumor malignancy [67]. Extensive studies have demonstrated that PDGF is indispensable for CAF function [68].

Platelets are the primary carriers of key proangiogenic factors, including VEGF, PDGF, and basic fibroblast growth factor (β-FGF). Among these, VEGF is one of the most potent angiogenic proteins transported and secreted by platelets [69]. Beyond their proangiogenic function, platelet–neutrophil interactions further contribute to tumor angiogenesis. Platelets become locally activated through the interaction of podoplanin with C-type lectin-like immune receptor 2 (CLEC-2) on tumor cells, as well as high-mobility group box 1 (HMGB1) with TLR4 [70,71]. Once activated, platelets release factors that promote cell adhesion and angiogenesis, enhancing tumor proliferation. In addition, they stimulate cytokine release from leukocytes, endothelial cells, and tumor cells, increasing local levels of IL-8, CXCL12, and CCL2, which support tumor cell proliferation and angiogenesis [72]. Interestingly, in addition to proangiogenic factors, platelets also serve as a source of antiangiogenic molecules, including thrombospondin 1 (TSP1), endostatin, and platelet factor 4 [73]. This suggests that stimulators and inhibitors of angiogenesis are tightly regulated and selectively released from distinct α-granules depending on specific stimuli. For instance, PAR1 activation selectively triggers the release of VEGF-containing granules, whereas PAR4 activation leads to the secretion of granules loaded with endostatin, but not VEGF [72].

Cancer cell-activated platelets play a pivotal role in tumor progression, particularly in metastasis. These platelets release mediators that regulate vascular permeability, thereby facilitating cancer cell extravasation and dissemination. Key mediators include eicosanoid metabolites such as TXA2 and 12-HETE, along with ATP, histamine, and serotonin. Their combined effects induce endothelial cell retraction, exposing the basement membrane and creating a permissive environment for cancer cell migration into distant tissues [74,75,76,77,78]. Furthermore, platelets contain enzymes capable of degrading extracellular matrix components, including matrix metalloproteinases (MMPs), platelet hyaluronidase 2, and heparinase [79,80,81,82]. Upon activation, these enzymes contribute to basement membrane degradation, facilitating tumor cell extravasation. Experimental studies have demonstrated that the intravenous injection of PDMP-conjugated cancer cells leads to an increase in pulmonary metastatic foci, supporting their role in metastatic spread [58]. Moreover, platelets co-localized with tumor cells secrete MMP-1/2/9, which degrades extracellular matrix components, weakening structural barriers and further promoting tumor invasion [83,84]. For example, in a murine model, platelet depletion in mice injected with B16/F10 melanoma cells resulted in reduced lung metastasis and significantly decreased MMP-2 and MMP-9 activity in extracellular microdialysate samples, suggesting a diminished capacity for extracellular matrix degradation and basement membrane transmigration at metastatic sites [85]. The involvement of PDGFs in epithelial–mesenchymal transition (EMT) has been demonstrated in multiple studies [86]. Platelets induce EMT in tumor cells through direct cell contact or by secreting transforming growth factor-beta (TGFβ), which activates the TGFβ/Smad and NF-κB signaling pathways [52]. For instance, cancer cells treated with TGFβ express PDGF-B, which is phosphorylated by platelets, leading to EMT. However, knocking out the TGFβ gene significantly reduces the metastatic potential of these tumor cells [87]. Similarly, PDGF released by highly activated platelets enhances MMP-2/9 expression and activates the p38/MAPK pathway, promoting EMT in platelet-stimulated cholangiocarcinoma cells and ultimately facilitating tumor dissemination and metastasis [88]. In addition to inducing EMT, platelets significantly modulate TME. Notably, platelet glycoprotein VI (GPVI) interacts with tumor-derived galectin 3 [89], integrin α6β1 binds to tumor ADAM9 [90], CLEC-2 recognizes tumor podoplanin [91], and GPIIb/IIIa associates with tumor ανβ3 integrin [92]. Together, these multifaceted interactions illustrate the dynamic and intricate relationship between platelets and tumor cells.

Beyond modifying the TME, platelets directly enhance cancer cell adhesion and arrest within the circulation through multiple adhesion molecules. Key platelet receptors such as GPIIb/IIIa, GPIb/IX, and GPVI mediate these interactions [93,94,95,96,97]. Experimental blockade or genetic deficiency of these receptors significantly reduces cancer cell adhesion and impairs metastatic progression in vitro, underscoring their crucial role in platelet–tumor interactions. Additionally, platelet-derived lysophosphatidic acid (LPA) stimulates cancer cells to produce IL-6 and IL-8, ultimately promoting osteolytic bone metastasis [98,99]. Cancer cell-conjugated platelets also coordinate the recruitment of other host cells during metastasis [100]. These platelets rapidly activate and release chemokines such as CXCL5 and CXCL7, which signal through the CXCR2 receptor to recruit granulocytes from the bloodstream to the platelet–cancer cell conjugate [100]. The co-localization of platelets and granulocytes aids in the formation of early metastatic niches, supporting cancer cell invasion and the establishment of metastatic foci [100].

## 5. Immunological Mechanisms and PLT in HCC

The role of platelets in immunological processes, both physiological and in the pathogenesis of inflammatory diseases and cancers, is increasingly being investigated. As morphological cells of blood, they can exist in two forms: at rest in the form of disk-like morphological cells and activated under the influence of vascular damage, but also, for example, bacterial antigens, as more spherical forms that are more susceptible to interaction with other cells. However, evidence has emerged demonstrating that under both resting and activated conditions, platelets possess a multitude of surface receptors and secrete various substances, thereby exerting a regulatory influence over the onset of immunological processes [101].

A number of advanced immunological processes involving platelets have been demonstrated; however, research in the context of HCC is currently underway and conclusions remain ambiguous. In particular, the interaction of PLTs with hepatic Kupffer cells may contribute to the inflammatory processes leading to cirrhosis and the secondary development of HCC. It has been demonstrated that Kupffer cells play a pivotal role in the recruitment of intrahepatic PLTs. Upon activation, PLTs release a broad range of mediators like serotonin, PDGF, and TGFβ, which can stimulate both Kupffer cells and hepatic stellate cells (HSCs), initiating a proinflammatory and pro-fibrotic cascade [102,103]. In turn, activated Kupffer cells secrete cytokines such as TNF-α and IL-1β, as well as ROS, further exacerbating hepatic injury and promoting chronic inflammation [104]. This sustained inflammatory microenvironment can contribute to extracellular matrix deposition and fibrotic remodeling, hallmark features of cirrhosis. Over time, such conditions create a permissive niche for malignant transformation, positioning the PLT–Kupffer cell axis as a critical tool in the progression from fibrosis to HCC [103].

Furthermore, an increase in chemokines and cytokines, which attract immune cells, has been observed in correlation with platelet activation. It is imperative to note that platelet adhesion and activation—but not aggregation—are essential for HCC. Moreover, in the context of repair mechanisms, the interaction between these cells and PLTs is imperative for the observed effects [102]. In general, it appears that PLTs interact with macrophages to induce immunosuppressive and pro-tumorigenic effects on the TME. It has been demonstrated that the production of TNFα is suppressed by PLTs, thereby impeding the antitumor capabilities of macrophages [105]. Tumor cells with high programmed death ligand 1 (PD-L1) expression can evade immune surveillance [106,107,108]. Zaslavsky et al. demonstrated the presence of PD-L1 in platelets from both healthy individuals and patients with advanced cancer using Western blot, confirming that PD-L1-expressing platelets contribute to overall PD-L1 expression in tumors [109,110,111]. Moreover, platelet attachment to PD-L1-negative tumor cells can induce PD-L1 expression, thereby facilitating immune evasion [111]. Platelets also influence T-cell activation. CD4+ and CD8+ T-cell activation is suppressed by platelet-derived TGFβ and lactate. Interestingly, studies using humanized mouse models have shown that platelet releases (PRs) completely suppress T-cell proliferation, blastogenesis, and interferon gamma (IFN-γ) production [112]. Research has further demonstrated that platelets regulate T-cell function, particularly in CD4+ T cells stimulated by CD3/CD28. Platelets produce cytokines such as IL-2, IFN-γ, and TNF-α, and they promote regulatory T-cell (T-reg) and Th17 differentiation via IL-10 and IL-17 secretion [113]. These immunomodulating effects are closely linked to tumorigenesis due to their immunosuppressive and proinflammatory functions [114]. The underlying mechanism by which platelets regulate immune responses involves both direct cell–cell contact and soluble mediators. Soluble mediators such as platelet factor 4 (PF4), TGFβ, and RANTES are released by platelets to modulate effector T-cell activation in platelet–T-cell co-cultures [113]. RANTES has been shown to enhance CD8+ T-helper cell cytotoxicity and cytokine production [115], while CXCL1 promotes Th17 differentiation and IL-17A production, which favors tumor growth [116,117]. Additionally, chemokines like CXCL4 and its non-allelic variant CXCL4L1, stored in platelet α-granules, exhibit chemotactic and angiostatic activity for leukocytes in the TME [118]. High concentrations of serotonin (5-HT) are stored in the dense granules of platelets, and T cells express a high density of 5-HT receptors. The translocation of nuclear factor kappa B (NFκB) to the nucleus is associated with T-cell survival and S-phase transition, driven by the stimulation of the 5-HT1A receptor [119]. Furthermore, serotonin and dopamine secreted from platelets enhance CD4+ T-cell proliferation and differentiation into the Th1 phenotype. Dopamine, acting as an immunomodulatory neurotransmitter, has a dual role: it stimulates naïve and resting T cells while suppressing activated T cells [120]. TGFβ secreted by tumor and stromal cells plays a role in tumor suppression by inducing cell-cycle arrest and apoptosis in non-cancerous epithelial cells during pre-cancerous stages [121,122]. This effect is mediated through the upregulation of cyclin-dependent kinase (CDK) inhibitors CDKN1A (p15), CDKN2B (p21), and CDKN1C (p57), as well as the apoptosis inducer death-associated protein kinase (DAPK). Additionally, TGFβ suppresses c-Myc, thereby exerting an inhibitory effect on tumor growth. Platelet-secreted TGFβ can also downregulate NKG2D, an activating receptor on natural killer (NK) cells, leading to reduced NK cell-mediated antitumor activity [123].

However, conflicting evidence exists regarding the role of platelets in tumor progression. For example, an experimental study demonstrated that platelets inhibited liver cancer growth in a mouse model of nonalcoholic fatty liver disease. This antitumor effect was mediated by P2Y12-dependent CD40L release, which activated CD8+ T cells via CD40 receptor signaling [124]. Additionally, platelets have been shown to inhibit tumor cell proliferation by inducing cell-cycle arrest at the G0/G1 phase [125]. During interactions between platelets and natural killer (NK) cells, tumor cells exploit platelet surface receptors to evade immune recognition, thereby enhancing their survival during early metastasis [126]. For example, platelets express “a disintegrin and metalloproteinase” (ADAM), with ADAM10 playing a role in the shedding of stress-induced NKG2D ligands, such as MICA/B and ULBP2, on tumor cells [127,128]. This mechanism facilitates tumor immune escape, further contributing to metastatic progression.

## 6. Thrombocytopenia and Thrombocytosis in HCC

Both elevated and decreased platelet counts are known to affect survival rates and recurrence in patients with HCC. Higher platelet counts were seen in patients with larger tumors [129], while low platelet counts were associated with smaller tumors [130]. Thrombocytopenia correlates with advanced fibrosis, portal hypertension, and higher MELD scores [129].

### 6.1. Thrombocytosis: Survival Rate and Recurrence

In most studies, thrombocytosis is defined as a platelet count above 300 × 10^9^/L [131,132], though this is often below standard cut-offs for normal platelet counts on complete blood count testing. High levels of platelet count are associated with worse outcomes in many tumors [133,134,135] and with higher thrombotic risk [136]. Increased platelet numbers may lead to enhanced availability of β-FGF and VEGF, which can induce neovascularization in addition to promoting metastases and evading the immune system via mechanisms described above.

A cohort study including Taiwanese and American populations found a significant difference between survival rates in patients with and without thrombocytosis. When compared, the Taiwan and US groups with HCC characterized by thrombocytosis had median OS of 6 and 4 months, respectively, compared to 32 and 14 months (HR 2.31, 95% CI 2.04–2.62) for patients with normal platelet levels. Furthermore, tumors in patients with thrombocytosis were characterized by greater vascular invasion (54% vs. 29% vs. 15% in the Taiwan cohort and 42% vs. 33% vs. 26% in the USA cohort) and increased lymph node involvement or extra-hepatic distant metastases (28% vs. 12% vs. 6% in the Taiwan cohort and 48% vs. 26% vs. 12% in the USA cohort) (all *p* < 0.001) [131].

In other analyses, high platelet level was associated with worse OS for HCC patients with BCLC stage B, with a nonlinear correlation observed between OS and PLT count, as the cut-off point for PLT > 67.6 × 10^9^/L significantly increased the risk of death among patients (HR 3.07; CI 1.91–4.92) [137]. Evaluation of liver and tumor-related prognostic factors for HCC patients in Child C cirrhosis showed that high platelet count (>8 × 10^4^/mm^3^) was associated with higher mortality. [138]. Also, high platelet counts are associated with early recurrence, CTCs, tumor size, BMI, AFP, presence of multiple lesions, and Ki-67 [134].

### 6.2. Thrombocytopenia: Survival Rate and Recurrence

In the case of thrombocytopenia, the cut-off value is harder to define, with a 2024 meta-analysis describing cut-offs ranging from less than 150 to fewer than 75 × 10^9^/L [139]. Platelets tend to decrease in liver disorders, with mechanisms including decreased thrombopoietin production as well as sequestration of platelets in enlarged spleen in those with cirrhosis and portal hypertension. Interestingly, while analyzing the liver parameters and PLT level, the worst survival rate was present in patients with both thrombocytopenia and relatively preserved liver functions [131].

Thrombocytopenia has been mentioned as a risk factor for lower OS and shorter RFS, as well as cardiopulmonary complications, high risk of reintubation, need for blood transfusion, septic complications, or renal insufficiency in patients undergoing surgery for HCC [140]. A retrospective analysis of 151 patients with HCC who underwent hepatectomy for HCC showed that a PLT count of 10^5^/mm^3^ and above was a factor for superior OS and RFS. Patients with a PLT count above or even this value were characterized with a lower HCC recurrence in contrast with patients with lower levels, with 66% and 89% recurrence rates (*p* = 0.009), respectively. A platelet count below this value also had a negative effect on OS, as the 5-year OS rate was 27% in this group versus 65% in the group with higher PLT levels (*p* < 0.001) [141]. Another retrospective study of 141 patients with HCC treated surgically showed that next to CRP and albumin levels, a platelet count of <120 × 10^3^/mm^3^ was an independent and significant poor prognostic factor [142]. A platelet count less than 150 × 10^3^/L was associated with worse long-term 5-year survival (HR 2.37, 95% CI: 1.07–5.24, *p* = 0.026) in those undergoing resection for HCCs smaller than 2 cm. [143]. In similar vein, Schrecker et al. retrospectively studied 128 patients who underwent surgical treatment of HCC and showed that low platelet count was associated with lower OS, with an HR of 1.25 per 50/nL decrease in platelet count (95% CI: 1.02–1.53, *p* = 0.034) and increased perioperative mortality [129]. This was also confirmed by another study by Taketomi et al., where a PLT level ≤ 150 × 10^3^/mm^3^ correlated with shorter disease-free survival (*p* = 0.0026) [144].

The effect of platelet count on prognosis appears to be independent of HCC treatment methods. In a study of 258 patients treated with radiofrequency ablation (RFA), the authors proved that a PLT count ≤ 10^5^/mm^3^ was associated with a higher risk of recurrence of HCC (HR 1.501, 95% CI: 1.076–2.094; *p* = 0.017) [145]. That can also be seen in another study comparing patients undergoing two different modes of treatment—surgery and RFA—where a PLT count below ≤10^5^/mm^3^ in patients undergoing RFA was associated with shorter RFS [146]. Interestingly, the 3- and 5-year survival rates of the resection (90.3% and 79.0%, respectively) and RFA groups were similar (87.4% and 74.8%). Another study of patients with solitary HCC who had undergone RFA showed that a PLT count below ≤10^4^/mm^3^ was found to be not only linked with worst RFS but also with distant recurrence and inferior OS (*p* for all <0.001) [147]. Another retrospective study concerning RFA in patients with HCC tumors ≤ 5 cm and compensated cirrhosis showed that PLT count, as in previous studies, was shown to be an independent risk factor of overall poor survival, recurrence, and risk of appearance of new lesions [148]. Treatment combining RFA with transcatheter arterial chemoembolization (TACE) for HCC also showed that a PLT count below 97 × 10^4^/mm^3^ was associated with poor OS (HR 1.87, 95% CI: 0.9764–3.585, *p* = 0.05) [149].

According to our meta-analysis [139], a PLT count below 100 × 10^9^/L increased the overall risk of death by 30% across all patient groups. These conclusions differed across treatment intention, as in individuals undergoing treatment with curative intent, this risk was amplified by 62%.

Recurrence rates also appear to be higher in patients with thrombocytopenia, with a meta-analysis of 17 studies [132] finding a platelet count of fewer than 100 × 10^9^/L a parameter predictive of recurrence. A recent 2025 HCC survival analysis of non-surgically treated patients proposed platelet count/spleen diameter ratio (PSL) as a novel predictive tool [150]. A PSL with a cut-off value of 909 was an independent factor for 3-year OS and PFS in HCC patients.

## 7. Clinical Application of Platelet-Directed Therapies in HCC

Recently, there has been growing interest in the potential role of antiplatelet therapy (APT) in preventing HCC, leading to a number of studies based on animal models and cell lines aimed at elucidating the molecular mechanisms that connect APT with HCC development. While the benefits of aspirin in preventing colorectal cancer are well established, an increasing body of evidence suggests that both aspirin and clopidogrel may also help prevent HCC through several biological mechanisms (Table 2) [151,152,153,154].

Specifically, aspirin inhibits the release of serotonin and cyclooxygenase 1 (COX-1), leading to the suppression of thromboxane A2. This suppression contributes to the creation of an anti-metastatic microenvironment that involves both endothelial and tumor cells [174,175,176,177]. On the other hand, clopidogrel works by inhibiting the expression of α-granule-stored proteins, such as P-selectin and CD40L, which are crucial in the transition from vascular injury to inflammation. These proteins play a significant role in heterotypic interactions between platelets, leukocytes, and the endothelium [174,177]. A recent study utilizing a murine model found that the P2Y12-inhibitor effect of clopidogrel reduces the number of tumor cells by promoting an antitumoral macrophage phenotype [178]. Investigation into the chemopreventive effects of aspirin in HCC has gained increasing attention. In a mouse model of chronic HBV immune-mediated HCC, Sitia et al. demonstrated that platelet activation contributes to liver inflammation and immune infiltration and that treatment with aspirin and/or clopidogrel reduced these effects and also reduced liver fibrosis and HCC progression without causing bleeding complications [155]. Li et al. also reported that aspirin enhanced the antiproliferative and apoptotic effects of IFNα in HCC cell lines, and in a nude mouse xenograft model, aspirin alone or in combination with IFNα reduced tumor growth and promoted apoptosis in tumor tissue expressing STAT1 [156]. It has also demonstrated that aspirin can modulate apoptotic signaling pathways in HCC. Aspirin treatment significantly upregulates the expression and activation of caspase 8 and caspase 9, key initiator caspases in the extrinsic and intrinsic apoptotic pathways, respectively. Activation of caspase 8 leads to the initiation of death receptor-mediated apoptosis, while caspase 9 activation promotes mitochondrial-mediated apoptosis. By enhancing the activity of these caspases, aspirin promotes apoptosis in HCC cells, contributing to the inhibition of tumor growth [156]. Moreover, Wang et al. found that aspirin reduced collagen deposition in the liver and delayed tumor growth by dampening the NFκB pathway [157].

From the bench to the bedside, several human studies provide supportive evidence that antiplatelet drugs could provide a protective effect in HCC. The first suggestion of a chemopreventive effect of aspirin in HCC came from the NIH-AARP (National Institutes of Health–American Association of Retired Persons) Diet and Health Study, which found that the recall of aspirin use was linked to a 41% lower risk of HCC and a 45% lower risk of mortality due to chronic liver disease (CLD) compared to those who did not use aspirin [158]. Additionally, the Liver Cancer Pooling Project reported that aspirin use, but not ibuprofen, was associated with a 32% reduced risk of HCC [159]. The reduction in HCC incidence was more pronounced with daily aspirin use and at lower doses (<163 mg), but was independent of the duration of treatment [159]. Hayashi et al. conducted a retrospective clinical study on 772 patients with hepatocellular carcinoma (HCC). The study evaluated the effect of common antiplatelet agents—specifically aspirin and clopidogrel—used after HCC diagnosis. Patients receiving these drugs showed better overall survival, slower tumor progression, and preserved liver function, suggesting a potential benefit of antiplatelet therapy in HCC treatment without increasing bleeding risk [153].

Simon et al. performed a large population-based cohort study in Sweden, analyzing over 50,000 patients with chronic hepatitis B or C. They found that low-dose aspirin use was associated with a significantly lower risk of HCC and reduced liver-related mortality over time. The protective effect increased with longer duration of aspirin use, without a significant rise in gastrointestinal bleeding [160]. A population-based study in Taiwan assessed the effects of antiplatelet therapy in patients with HBV-related HCC undergoing liver resection. This study found that aspirin and/or clopidogrel use (with 88% receiving only low-dose aspirin) was associated with a 23% reduction in HCC recurrence and a 43% decrease in overall mortality compared to nonuse. However, the use of antiplatelet therapy was also linked to a 90% increased risk of upper GI bleeding. Despite these benefits, the mean duration of therapy was 1.3 years, with a median follow-up of 3.9 years, which was shorter than in other study populations [161]. Similarly, the Taiwanese Veterans study reported that aspirin use for cardiovascular prevention for at least 30 days before tumor resection led to an 82% reduction in HCC recurrence, although it did not influence overall survival in HBV-infected patients undergoing curative resection [162]. Other studies have further reinforced these findings. A pooled analysis of the Nurses’ Health Study (NHS) and the Health Professionals Follow-up Study (HPFS) revealed that regular aspirin use was linked to a 49% reduction in HCC incidence in both men and women. The protective effect was dose- and duration-dependent, becoming evident after five years of using at least 1.5 standard-dose (325 mg) aspirin tablets per week [163]. Additionally, a study from the National Cohort Study of Korean Adults, which involved a high-risk population due to endemic HBV infection, found that aspirin use was associated with a 13% reduction in HCC risk (*p* trend = 0.002), but did not significantly reduce HCC-related mortality. However, the combination of aspirin with non-aspirin NSAIDs amplified the chemopreventive effect, reducing the HCC risk by 35% compared to non-users [164]. Aspirin’s potential as an adjuvant therapy has also been explored. In a meta-analysis of six retrospective cohort studies, aspirin was shown to reduce HCC recurrence by 26% and all-cause mortality by 41% in patients undergoing liver resection or transarterial chemoembolization [179]. Furthermore, aspirin has been suggested to synergize with other treatments for advanced HCC. A recent retrospective study indicated that combining aspirin with sorafenib, an FDA-approved protein kinase inhibitor, led to prolonged overall survival in patients with advanced HCC [152]. HCC cells react to anticancer drugs like sorafenib and doxorubicin [166,169,170,171] and reduce the pro-metastatic effects of sorafenib [167,168].

Despite these promising results, aspirin therapy is not without risks, particularly in cirrhotic patients who are at increased risk of bleeding. The toxicity of aspirin was assessed in several retrospective studies, revealing divergent results regarding bleeding risks [165,173]. In particular, aspirin’s potential to increase GI bleeding in patients with cirrhosis remains a major concern. In addition, hepatorenal syndrome could be triggered by aspirin, which induces renal vasoconstriction and reduces the glomerular filtration rate [172]. This suggests that aspirin may not be suitable for all patients, especially those with advanced liver disease. Despite the positive outcomes observed in several studies, there are still limitations to retrospective studies in the general population, such as non-randomized treatment allocation, variable dosages and durations of therapy, and confounding factors like age, sex, cirrhosis status, and HBV/HCV infection. These factors complicate the interpretation of results and could introduce bias. An ongoing clinical trial is investigating aspirin in combination with the nucleoside analogue lamivudine in HBV patients after surgical resection (NCT01936233). In conclusion, while aspirin has shown potential benefits in reducing HCC recurrence and mortality, especially when combined with other treatments, its safety profile in cirrhotic patients remains a concern, particularly with the risk of GI bleeding and renal complications. Further clinical studies are needed to refine treatment protocols and determine the optimal duration of aspirin therapy to achieve the best clinical outcomes in HCC prevention and treatment.

## 8. Conclusions

A growing body of evidence has emerged supporting the role of platelets not only in physiological and regenerative processes in the liver but also in mechanisms of proliferation and remodeling associated with neoplasia and HCC pathogenesis. As an active element of the TME, platelets release mediators that are key to creating an inflammatory environment. They also participate in various intercellular interactions between tumor cells and elements of the immune system or other stromal cells via surface receptors. PLTs release mediators, including VEGF, TGFβ1, PDGF, serotonin, interleukins, and chemokines, which induce a proliferative effect by suppressing specific genes or activating transcription factors. Platelets may also contribute to the formation of metastases due to the large amount of angiogenic factors present in their intracellular vesicles, released upon their activation, and the remodeling of the environment.

In contemporary clinical studies, considerable emphasis has been placed on the prognostic and predictive effect of both platelet number and function on those with HCC. A comprehensive review of clinical observations indicates that the platelet count closest to normal is associated with the most favorable prognosis.

In the future, the primary emphasis should be placed on randomized clinical trials evaluating antiplatelet therapy as concomitant treatment in a particularly selected group of patients with hepatocellular carcinoma.

## Figures and Tables

**Table 1:** Overview of factors released from platelets upon activation.

Class of Substances	Substance
Adhesion proteins	Fibrinogen Fibronectin Thrombospondin Vitronectin von Willebrand factor
Coagulation factors	Factor V Factor VII Factor XI Factor XIII Kininogens Plasminogen Protein S
Protease inhibitors	C1 inhibitor Plasminogen activator inhibitor 1 Platelet inhibitor of factor XIα Platelet-derived collagenase inhibitor Protease nexin-II/amyloid β-protein precursor Tissue factor pathway inhibitor α-1-proteinase inhibitor α_2_-antiplasmin α_2_-antitrypsin α_2_-macroglobulin
Proteoglycans	Histidine-rich glycoprotein Neutrophil-activating peptide 2 Platelet basic protein Serglycin
Chemokines	CXC chemokine ligand (CXCL) 1, 2, 3, 4, 5, 6, 7, 8, 12, and 16 Macrophage inflammatory protein 1α Neutrophil-activating peptide 2 Platelet factor 4 Regulated upon activation, normal T-cell expressed and presumably secreted (RANTES)
Amines	Adrenaline Dopamine Histamine Noradrenaline Serotonin
Growth factors	Basic fibroblast growth factor Endothelial cell growth factor Epidermal growth factor Hepatocyte growth factor Insulin-like growth factor 1 Interleukin 2, 3, 4, 6, 7, and 8 Interleukin β Platelet-derived growth factor Transforming growth factor β Vascular endothelial growth factor A and C

**Table 2:** Summary of studies on antiplatelet therapy and hepatocellular carcinoma (HCC).

Study/Author	Reference	Model/Population	Findings	Notes
Sitia et al.	[155]	Mouse model (chronic HBV immune-mediated HCC)	Aspirin ± clopidogrel reduced liver inflammation, fibrosis, and HCC progression	No bleeding complications
Li et al.	[156]	HCC cell lines and mouse xenografts	Aspirin enhanced IFNα’s antiproliferative/apoptotic effects and reduced tumor growth	STAT1 expression important
Wang et al.	[157]	Mouse model	Aspirin reduced collagen deposition and slowed tumor growth via NFκB pathway inhibition	—
NIH-AARP Study	[158]	US cohort (recall-based)	Aspirin use is linked to a 41% lower HCC risk and a 45% lower CLD mortality	Observational, recall bias is possible
Liver Cancer Pooling Project	[159]	Meta-analysis	Aspirin (not ibuprofen) is associated with a 32% lower HCC risk	The effect is stronger with daily low doses (<163 mg), independent of duration
Retrospective	[153]	772 HCC patients	Antiplatelets (aspirin, clopidogrel) linked to better survival, slower progression	No increased bleeding risk
Cohort Study	[160]	50,000+ chronic hepatitis B/C patients (Sweden)	Low-dose aspirin is associated with lower HCC risk and mortality	Protective effect increased with longer use; no GI bleeding increase
Taiwan HBV Cohort	[161]	HBV-related HCC post-liver resection	APT (mostly low-dose aspirin) reduced recurrence (23%) and mortality (43%) but increased GI bleeding risk (90%)	Median follow-up: 3.9 years
Taiwanese Veterans Study	[162]	HBV patients undergoing resection	Aspirin use ≥30 days pre-surgery reduced HCC recurrence by 82%	No effect on overall survival
NHS & HFS	[163]	US cohort (men and women)	Regular aspirin use reduced HCC incidence by 49%	Dose- and duration-dependent effect (≥1.5 tabs/week for ≥5 years)
Korean Cohort	[164]	High HBV risk population	Aspirin use reduced HCC risk by 13% but not mortality	Combined with NSAIDs: 35% HCC risk reduction
Meta-analysis	[165]	6 retrospective cohort studies	Aspirin reduced recurrence by 26% and all-cause mortality by 41%	Liver resection or TACE for patients
Retrospective study	[152]	Advanced HCC patients	Aspirin + sorafenib improved overall survival	Suggests synergy with targeted therapy
Preclinical studies	[166,167,168,169,170,171]	In vitro/in vivo	Aspirin-sensitized HCC cells to sorafenib/doxorubicin and reduced metastasis	Mechanistic studies
Safety studies	[165,172,173]	Retrospective	Mixed results on bleeding risk, especially in cirrhotics	Aspirin may worsen renal function (hepatorenal syndrome)
Ongoing Trial	NCT01936233	HBV patients post-resection	Evaluating aspirin + lamivudine	Randomized trial to provide more robust data

APT—antiplatelet therapy; CLD—chronic liver disease; GI—gastrointestinal; HBV—hepatitis B virus; HFS—Health and Family Service; IFN-α—interferon α; NFκB—nuclear factor kappa light-chain enhancer of activated B cells; NHS—National Health Service; NSAID—nonsteroidal anti-inflammatory drug; STAT1—signal transducer and activator of transcription 1; TACE—transarterial chemoembolization; US—United States.

## Data Availability

Not applicable.
